# Rapid Quantification of Myocardial Lipid Content in Humans Using Single Breath-Hold ^1^H MRS at 3 Tesla

**DOI:** 10.1002/mrm.23011

**Published:** 2011-06-30

**Authors:** Belen Rial, Matthew D Robson, Stefan Neubauer, Jürgen E Schneider

**Affiliations:** Department of Cardiovascular Medicine, University of OxfordOxford, United Kingdom

**Keywords:** cardiac magnetic resonance spectroscopy, breath-holding, 3 T, lipid, quantification

## Abstract

A rapid, proton magnetic resonance spectroscopy method to evaluate human myocardial lipid levels in a single breath-hold at 3 T using a commercial whole-body system is presented. During a 10 s breath-hold, water unsuppressed and suppressed spectra were acquired by two phased array coils using a short-echo time spectroscopic stimulated echo (STEAM) sequence electrocardiogram-triggered to mid-diastole. Lipid-to-water ratios were obtained in the septum of 15 healthy volunteers, (0.46 ± 0.19)%. These results agreed well with ratios obtained from averaged spectra acquired in seven multiple breath-holds, (0.45 ± 0.20)%, providing increased signal-to-noise ratio but requiring longer acquisition times. Excellent correlation was found between the two methods (*r* = 0.94, *P* < 0.05). Reproducibility of ^1^H MRS for measuring myocardial lipid levels in a short breath-hold was acceptable in five repeated measurements within the same subject (coefficient of variation = 19%). Thus, single breath-hold proton spectroscopy allows reliable and quick quantification of myocardial lipids at 3 T. Magn Reson Med, 2011. © 2011 Wiley-Liss, Inc.

Recent advances in cardiac proton magnetic resonance spectroscopy (^1^H MRS) have enabled the noninvasive study of myocardial lipid metabolism in humans (1–3) typically requiring a 4–10 min acquisition. Initial studies in humans have confirmed findings from animal research, suggesting that cardiac lipid levels may be considered as a potential biomarker for myocardial dysfunction (4–7). Therefore, a further characterization of the pathological role of myocardial lipid deposits may benefit from the application of a noninvasive tool such as ^1^H MRS.

The acquisition of high-quality cardiac proton spectra is technically demanding. The signal-to-noise ratio (SNR) for myocardial metabolites is low because of their low concentrations and due to the significant distance from the radiofrequency (RF) coils. Nowadays, clinical 3 T scanners with phased-array receive coils are increasingly common, with the advantages of increased SNR (8) and higher spectral resolution. However, high-field MR spectroscopy suffers from increased magnetic field inhomogeneities (9) that pose further challenges for the water signal suppression, essential to detect the weak metabolite signals. Moreover, cardiac and respiratory motion results in changing voxel location and can, therefore, affect shimming and water suppression. Electrocardiogram gating is generally adequate for compensating cardiac motion. Various approaches have been proposed to reduce the influence of respiratory motion, including respiratory gating (1, 2, 10) and navigator gating with volume tracking (3, 11, 12). Despite the progress of cardiac ^1^H MRS as a research tool, only few studies so far have examined cardiac metabolism in humans using this tool at 3 T (11–13).

Here, a single breath-hold cardiac-gated ^1^H MRS acquisition is proposed as a time-efficient approach to measure myocardial lipid levels at 3 T. Breath-holding is commonly used for respiratory motion compensation in thoracic MR imaging, with comfortable end-expiration breath-hold periods limited to 25 s for healthy volunteers and 9 s for patients (14, 15). Hence, the aim of this study was to investigate whether cardiac lipid levels can be measured accurately within a single breath-hold using ^1^H MRS at 3 T, and to evaluate the applicability and reproducibility of this method as a routine clinical research tool.

## MATERIALS AND METHODS

### Subjects

In 15 healthy volunteers (11 men, four women; mean age ± standard deviation (SD), 34 ± 10 years; age range, 22–58 years; body mass index (BMI), 19–30 kg/m^2^), myocardial lipid normalized to myocardial water content (myocardial lipid levels) were evaluated using ^1^H MRS. The volunteers were asked to fast overnight. None of the volunteers had a history of cardiovascular disease, diabetes, or any other chronic disease. The Research Ethics Committee at our institution approved the study protocol, and all participants gave informed consent.

### ^1^H MRS Technique

All studies were performed on a 3 T MR scanner (Tim Trio, Siemens Healthcare, Germany). The ^1^H MRS sequence was based on a conventional STEAM sequence that was modified to achieve a short TE of 10 ms. Water suppression was achieved using a WET module (16). The standard prescan approach was used for adjusting the RF pulse scaling factor, which utilizes a subject dependent global calibration. Additionally, a calibration pulse sequence was implemented to evaluate the optimal water suppression pulse scaling factor. Cardiac gated spectra were acquired using different water correction scaling factors in a 17 s breath-hold and the one yielding the most effective water-suppression was chosen and applied in the following experiments.

**FIG. 1:**
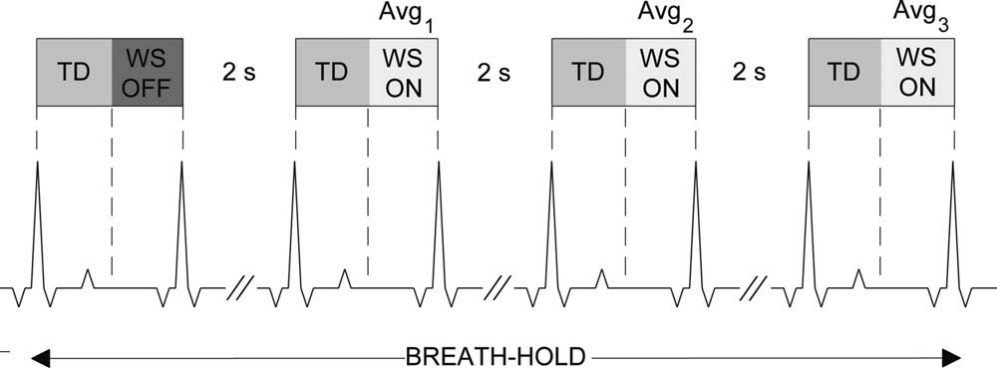
Single breath-hold acquisition scheme: a water-unsuppressed scan (WS OFF) was followed by 3 water-suppressed scans (WS ON), with a 2-s delay in between acquisitions. Each scan was cardiac gated, and a trigger delay (TD = 635 ± 82 ms, mean ± SD, *n* = 15) was used to position each STEAM module at the same time in mid-diastole for both WSON and WSOFF. The WET module (duration: 218 ms) was applied with the amplitude of the RF pulses set to 0 during the WSOFF scan.

During the single breath-hold acquisition, the modified STEAM sequence was repeated four times: a spectrum without water suppression was obtained first, with the frequency centred at the water frequency followed by, three consecutive water-suppressed measurements with the frequency set to 3 ppm (Fig. 1). The effective repetition time (TR) was cardiac gated and controlled by the pulse program to be at least 2 s and the total spectroscopy acquisition time was less than 10 s. To determine whether or not the single breath-hold technique provided sufficient SNR for an accurate myocardial lipid assessment, it was compared with the multiple breath-hold method. This consisted of six breath-holds of about 16 s each, five breath-holds which allowed for the acquisition of 35 nonaveraged water-suppressed spectra. Also four nonaveraged water spectra were acquired with a minimum TR of 4 s in a separate breath-hold setting the water suppression RF pulse power to zero. The total acquisition time was 5–7 min, including time for the volunteers to recover between breath-holds.

### Validation of Single Breath-Hold Lipid Content Measurement

**FIG. 2:**
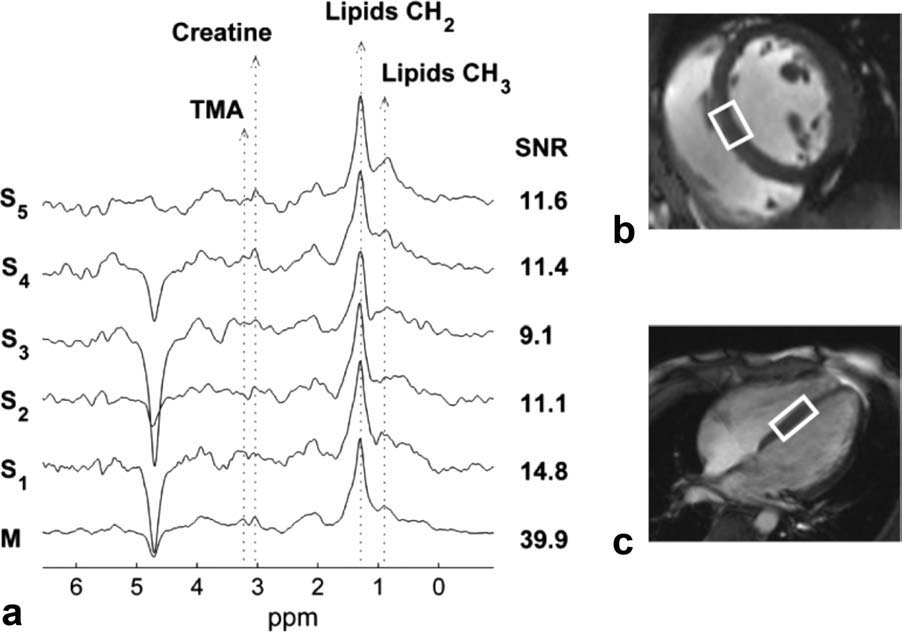
**a:** Illustration of reproducibility of single breath-hold ^1^H MRS measurements compare with multiple breath-hold measurements. Within subject reproducibility was demonstrated by five repeated spectra (S_1_-S_5_) acquired in the same session from a 22x12x32 mm voxel positioned in the interventricular septum of a healthy volunteer (male, BMI = 28 kg/m^2^) as shown in (**b**) and (**c**). Depiction of the myocardial lipid resonance (-CH_2_)_n_, at 1.3 ppm obtained in a single breath-hold (3 averages, myocardial lipid content: 0.61%) showed sufficient SNR as confirmed by almost the same spectrum (M) acquired using multiple breath-hold ^1^H MRS (35 averages, myocardial lipid content: 0.64%). The spectra were scaled to the respective water amplitude.

For the validation of the method, the volunteers were positioned supine, the body coil was used for transmission and the anterior and posterior phased-array body coils for receiving, resulting in 15 channels of data. Cardiac gated MR cine imaging was performed to acquire the standardized four-chamber and short axis views for appropriate voxel placement in the septum and for determination of the respective trigger delay required for mid-diastole (Fig. 2b,c). Shim adjustment was performed on a 3D field map obtained from a cardiac-gated gradient double echo acquisition within a single breath-hold (18–22 s). Myocardial ^1^H MRS data were obtained at end-expiration from a 22 × 12–19 × 32–36 mm (8–15 mL) voxel centered in the interventricular septum far from epicardial fat (Fig. 2). All acquisitions were electrocardiogram-triggered to mid-diastole. Spectroscopy parameters for the custom STEAM sequence included a TE of 10 ms, a mixing time of 7 ms, 1024 points were acquired at a bandwidth of 2000 Hz. Effective repetition times of at least 4 and 2 s were chosen to approach complete relaxation of the water and lipid signals, respectively. Furthermore, the scan frequency was set at 4.7 ppm during water-unsuppressed acquisitions and at 3 ppm during water-suppressed acquisitions, to minimize the effects of the large chemical shift displacement (≍ 420 Hz corresponding to 4.5 mm in the feet to head direction) between the lipid and water peaks at this field strength.

To investigate the reproducibility of the single breath-hold method, this technique was repeated five times in each subject without repositioning the voxel. To assess the accuracy of the single breath-hold method, the multiple breath-hold method was applied at an identical voxel location. Imaging and prescan adjustments were repeated again before multiple breath-hold acquisitions. Total session time comprising positioning of the patient, septum localization, shimming, water suppression factor adjustment, and acquisition of a single breath-hold spectrum was on average 15 min. This time was increased by a further 5–7 min for the multiple breath-hold spectra acquisition.

### Spectral Postprocessing

The algorithms for signal combination from individual coil elements (17) and averaging from different acquisitions were written in Matlab. The combination of the individual signals was performed using the amplitude and phase of the time-domain signal without water suppression to represent the weighting and phase correction factors required for weighted signal summation. The multiple ^1^H MRS acquisitions (i.e., three during single breath-hold measurements and 35 during multiple breath-holds measurements) were phase-corrected with the zero-order phase of the dominant peak in the spectra (typically the residual water peak or the lipid peak) before averaging (12). Furthermore, individual signals acquired within different breath-holds were frequency aligned prior to the summation.

All spectral quantification was performed in the time domain, using the AMARES algorithm included in the jMRUI package (18). A total of six peaks were specified to fit the metabolite contributions in the water-suppressed spectra by using a Lorentzian line shape, representing total creatine with methylene group CH_2_ at 3.9 ppm, trimethylammonium compounds at 3.2 ppm, total creatine with methyl group CH_3_ at 3.0 ppm (Cr), lipids at 2.2 ppm, lipids (-CH_2_) at 1.3 ppm, and lipids with methyl group CH_3_ at 0.9 ppm. The prior knowledge applied was a restriction of linewidths to below 40 Hz for all resonances and only the global zero-order phase was fitted, while the first-order term was kept constant. The amplitude of the lipid resonance at 1.3 ppm was selected for myocardial lipid quantification. The water peak amplitude from the water-unsuppressed scans was used as internal reference. The lipid content was calculated as a percentage relative to water as the amplitude of the lipid peak divided by the amplitude of the water peak, and multiplied by 100. The SNR of the lipid peak was estimated by taking the ratio of the lipid time domain signal intensity to the noise SD extracted from the final 100 points of the signal in the time domain. Also, an estimate of the SNR was obtained by dividing the Cramer-Rao standard deviation (CRSD) of the lipid peak, which is an indicator of the accuracy of the spectral quantification provided by the AMARES fitting algorithm, by the lipid peak amplitude and converted to a percentage (rCRSD%) (19).

### Statistical Analysis

Statistical analyses were performed using SPSS (version 16.0; Chicago, IL). Results were expressed as mean ± SD. To determine the reproducibility of the technique, coefficients of variation (CV) of the five repeated myocardial lipid content measurements in a single breath-hold were calculated as CV = (within-subject SD)/(within-subject mean of five myocardial lipid% measurements) × 100. The Pearson correlation coefficient (*r*) and linear regression analysis were used to examine the relationship between myocardial lipid content measured in a single breath-hold and multiple breath-holds and between myocardial lipid content and BMI. Bland-Altman analysis was performed to evaluate the agreement between myocardial lipid levels by single and multiple breath-holds measurements. A paired *t*-test was used to compare myocardial lipid content, CRSD% and linewidth values between the two acquisition approaches. A *P* < 0.05 was considered statistically significant.

## RESULTS

Representative spectra from the septum of the healthy volunteers using both, single and multiple breath-hold methods are shown in Fig. 2a. The spectra showed a well-defined resonance corresponding to myocardial lipids at 1.3 ppm, relative to the residual water at 4.7 ppm. Other resonances, such as trimethylammonium at 3.2 ppm, creatine at 3 ppm, and other lipids at 0.9–2.5 ppm were also visible in the multiple breath-hold spectra.

Constructive averaging of the 35 STEAM spectra acquired using the multiple breath-hold method resulted in the expected increase in SNR and lower lipid rCRSD% compared with the single breath-hold method (SNR: 24 ± 14 vs. 6.8 ± 4.2, *P* < 0.05; rCRSD%: 4.4 ± 3.4 vs. 9.1 ± 5.9, *P* < 0.05; multi vs. single breath-hold). In the 35 STEAM spectra acquired using the multiple breath-hold method, the mean phase variation for all subjects was 26 ± 19° (mean ± SD, *n* = 15). The water full-width-at-half-maximum in the unsuppressed water spectra was 14.3 ± 3.2 Hz and 13.3 ± 2.4 Hz (*P* = 0.38) when using the single breath-hold method (one average) and the multiple breath-hold method (four averages), respectively.

**FIG. 3:**
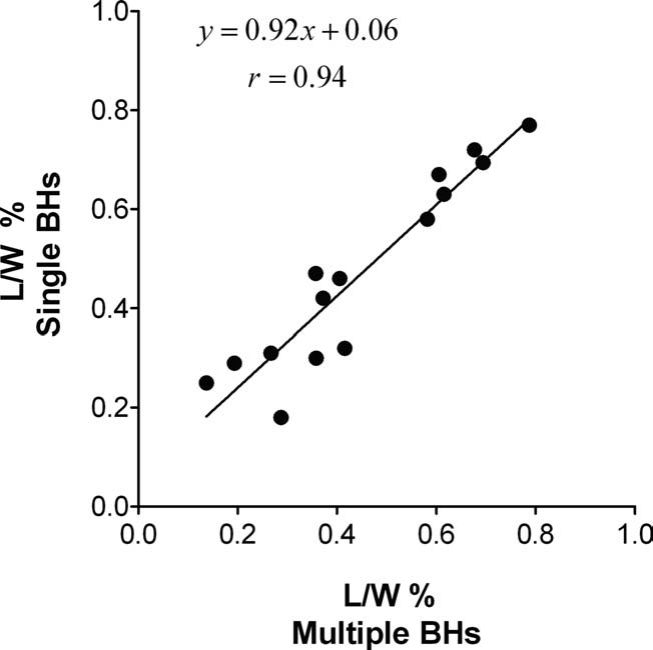
Linear regression analysis showing the strong correlation between myocardial lipid content measured relative to water in one breath-hold and multiple breath-holds (*P* < 0.05). A single breath-hold spectrum out of the five repeated scans was randomly selected for each subject.

**FIG. 4:**
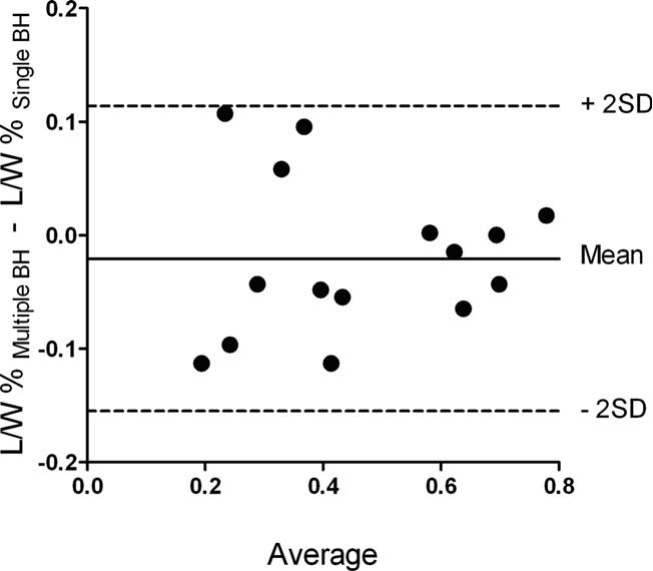
Bland-Altman plot for myocardial lipid levels acquired using single and multiple breath-holds methods. The single breath-hold data were randomly chosen from the 5 repeated measurements. The solid line represents the mean of differences between levels obtained with different methods and the dashed lines indicate the confidence intervals ± 2 SD.

The mean myocardial lipid content in healthy volunteers with both single and multiple breath-hold ^1^H MRS was (0.46 ± 0.19)% and (0.45 ± 0.20)%, respectively (*P* = 0.94). Within-subject reproducibility of myocardial lipid levels over repeated ^1^H MRS measurements in a single breath-hold showed a CV of 19%. Moreover, a strong correlation was confirmed between myocardial lipid levels obtained in a single breath-hold (randomly selected from the five repeated single breath-hold measurements) and multiple breath-holds (*r* = 0.94 *P* < 0.05, Fig. 3). Bland-Altman analysis (Fig. 4) showed an excellent agreement of the lipid levels measured in a single breath-hold and in multiple-breath-holds, with a mean difference between both methods of (−0.02 ± 0.07)%. All the differences between the two measurement methods were contained within the 95% limits of agreement (from −0.13% to 0.11%).

**FIG. 5:**
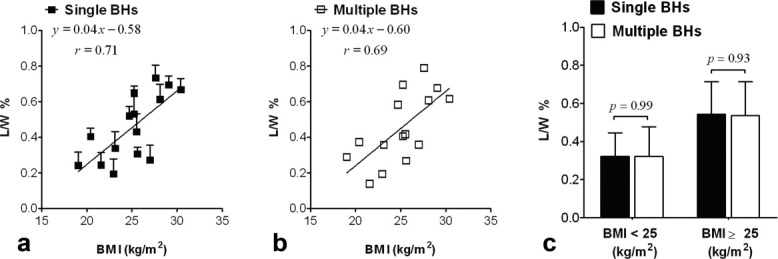
Myocardial lipid levels correlated positively with BMI for both single (**a**) and multiple (**b**) breath-holds acquisitions. The error bars in panel (a) represent the SD of the five repeated scans using the single breath-hold method. Volunteers with BMI ≥ 25 kg/m^2^ showed myocardial lipid levels significantly higher than volunteers with BMI < 25 kg/m^2^ (*P* < 0.05). No significant difference was found between methods for both BMI groups.

For the BMI range represented in this study, the myocardial lipid levels increased linearly with BMI (Fig. 5a, single breath-hold: *r* = 0.71, *P* < 0.05; Fig. 5b, multiple breath-holds: *r* = 0.68, *P* < 0.05). Moreover, the difference between the slopes of regression lines for both methods was not different (*P* = 0.98). The 15 volunteers were then divided into two groups: normal (BMI < 25 kg/m^2^, four male, two female) and overweight (BMI ≥ 25 kg/m^2^, seven male, two female). Myocardial lipid levels were significantly different between the two groups and independent of the method of measurement used (Fig. 5c, single breath-hold: lipid levels lean group = (0.32 ± 0.12)%, lipid levels overweight group = (0.54 ± 0.17)%, *P* = 0.02; multiple breath-hold: lipid levels lean group = (0.32 ± 0.16)%, lipid levels overweight group = (0.54 ± 0.18)%, *P* = 0.03).

## DISCUSSION

This study has shown that myocardial lipid levels can be quantified during a short single breath-hold using cardiac triggered proton spectroscopy at 3 T. The mean lipid levels obtained using single and multiple breath-hold methods agreed well. Water was chosen as the internal reference as it is assumed that the water content remains constant in normal and pathological conditions as shown by Bottomley and Weiss (20) in the dog model of myocardial infarction. Other metabolites, such as creatine not only suffer from low SNR but may also vary in pathologies.

The spectroscopic quality, characterized by the linewidth of the unsuppressed water signal (14.3 ± 3.2 Hz), was good using the single breath-hold method and similar to values published at 1.5 T. van der Meer et al. (3) reported a myocardial water signal linewidth of 10.3 Hz using navigator gating and volume tracking for respiratory motion correction and 11.4 Hz without navigation. Other study obtained a 12 Hz myocardial water linewidth using a sequence gated to the respiratory cycle (21). Because linewidth is dependent on the T_2_-relaxation time and field homogeneity, the linewidth would be expected to increase as the field strength goes from 1.5 T to 3 T. The similar values were attributed to the good local shimming achieved at 3 T in this study that used a cardiac gated 3D field map shimming method that controlled both the 1st and 2nd order shim coils. Furthermore, the CRSD was on average 9.1% of the myocardial lipids demonstrating that single breath-hold acquisitions showed sufficient spectral quality (22) over the wide range of myocardial lipid levels observed (0.14–0.73%). This finding is also supported by the fact that an almost identical myocardial lipid range (0.14–0.79%) was observed using multiple breath-holds acquisition, with intrinsically lower CRSD (4.4%).

The reproducibility of the single breath-hold method was validated by repeating single breath-hold lipid measurements at the same myocardial location for each subject. Although the volunteers were not repositioned in our reproducibility assessment, all the reference scans (i.e., scouting, frequency adjustment, RF-reference voltage measurement, shim current, and water suppression calibration) were invalidated and forced to be re-calibrated in between measurements. In our experience, the calibration procedure rather than the patient positioning represent the main source of variation for this kind of measurements (due to breath-hold variability). A CV = 19% was obtained. Szczepaniak et al. (1) showed similar CV (17%) for spectroscopy measurements of myocardial lipid levels using a pressure belt for respiratory gating. Felblinger reported a CV of 13% using double triggering based on the electrocardiogram signal (10). Also, findings in this study compared well with more recent results obtained at 1.5 T using navigator gating and volume tracking (3).

Volunteers were divided into two groups according to their BMI. The threshold of 25 kg/m^2^ was based on the National Institutes of Health classification of overweight by BMI (23). Importantly, single breath-hold ^1^H MRS showed sufficient SNR for the assessment of cardiac lipid levels, even for low, i.e., normal BMI volunteers. Moreover, a statistically significant positive correlation between BMI and myocardial lipid levels was found, which was also reported in previous studies where other methods were used for spectroscopy acquisition (1, 2, 24).

This study demonstrated the time efficiency obtainable from single breath-hold MR acquisitions. In a 10 s breath-hold, both unsuppressed and suppressed water signals were acquired with sufficient resolution for myocardial lipid levels quantification in healthy volunteers. Compared with multiple breath-hold averaging, single breath-hold ^1^H MRS acquisition time decreased from 16 to 10 s, which allowed for a comfortable breath-hold and more importantly can be applied to patients, who can generally hold their breath only for shorter times compared to volunteers (15).

No T_1_ corrections were considered in the calculation of the metabolite ratios. The T_1_ of lipids was to be between 300 and 400 ms (estimated based on values reported for 1.5 T (25)). Hence, the TR of 2 s was adequate to ensure full relaxation. The T_1_-value for healthy myocardium was measured to be 1.2 s (26). Although the water signal was not fully relaxed at a TR of 4 s, the correction factor of 3% is within the accuracy of the method. Importantly, water was fully relaxed for the single-breath-hold method. Furthermore, the lipid level values obtained in this study were in concordance with previous published data (3, 5, 13, 27).

The spectroscopy voxel was larger (8–15 mL) compared with previous studies (2–9 mL) (1, 3, 13, 20, 24, 28) However, contamination from ventricular blood can be excluded due to the dark blood properties of the STEAM sequence (20, 28, 29), which we confirmed in preliminary work by placing the voxel entirely in the blood pool (data not shown). Moreover, the spectroscopic volume was carefully positioned in the interventricular septal wall, avoiding areas of pericardial fat, confirmed by a single spectral peak at 1.3 ppm (1). The water-suppressed localized volume was based on the creatine resonance frequency (3 ppm) for possible assessment of the entire myocardial metabolic range, including creatine. For these experimental settings (≍3.4 kHz STEAM sinc refocusing pulses, length = 2.6 ms and voxel volume = 8–15 mL), the creatine and lipid resonances had ≍ 82% of their corresponding voxels in common. A creatine peak was clearly visible in 12 of the 15 multiple breath-hold spectra acquired. However, creatine was not quantified in these spectra as the aim of this study was to establish and validate the single breath-hold technique. Furthermore, single breath-hold cardiac spectra did not have sufficient SNR for reliable creatine quantification. Moreover, future single breath-hold experiments should be acquired at the lipid resonance.

Although only 15 volunteers were included in this study to investigate feasibility and to validate single breath-hold cardiac spectroscopy, the sample size was sufficient to achieve statistical significance by single breath-hold and multiple breath-hold lipid levels correlating with a slope of unity. However, ^1^H MRS was only performed in healthy volunteers with a BMI range 19–30 kg/m^2^. Hence, the performance of the technique specifically in obese patients requires further investigation. Notably, a 10 s breath-hold is well within the comfortable range for healthy volunteers (14) and also for patients (15). A breath-hold period of similar duration is routinely used for functional cardiac MR imaging in our institution. Importantly, the minimal time requirement of the proposed protocol combined with the use of standard cardiac receive coils allows this method to be added to any routine cardiac MR exam without substantial increase of scan time.

## CONCLUSION

Single breath-hold 3 T ^1^H MRS is a feasible and reproducible method for the measurement of cardiac lipid levels in a spectrum varying from low to more elevated cardiac lipid levels in healthy volunteers. Additionally, this is a quick tool to better characterize the influence of cardiac lipid accumulation on cardiac function or to evaluate the effects of pharmacological or dietary interventions.
